# Fathers matter: male body mass affects life-history traits in a size-dimorphic seabird

**DOI:** 10.1098/rspb.2017.0397

**Published:** 2017-05-03

**Authors:** Tina Cornioley, Stéphanie Jenouvrier, Luca Börger, Henri Weimerskirch, Arpat Ozgul

**Affiliations:** 1 Department of Evolutionary Biology and Environmental Studies, University of Zurich, Winterthurerstrasse 190, 8057 Zurich, Switzerland; 2 Woods Hole Oceanographic Institution, Mailstop 50, Woods Hole, MA 02543, USA; 3 Centre d'Etudes Biologiques de Chizé, Station d'Ecologie de Chizé-La Rochelle, CNRS UMR 7372, 79360 Villiers-en-Bois, France; 4 Department of Biosciences, College of Science, Swansea University, Singleton Park, Swansea, Wales SA2 8PP, UK

**Keywords:** wandering albatross, bi-parental care, sexual dimorphism, survival, reproduction

## Abstract

One of the predicted consequences of climate change is a shift in body mass distributions within animal populations. Yet body mass, an important component of the physiological state of an organism, can affect key life-history traits and consequently population dynamics. Over the past decades, the wandering albatross—a pelagic seabird providing bi-parental care with marked sexual size dimorphism—has exhibited an increase in average body mass and breeding success in parallel with experiencing increasing wind speeds. To assess the impact of these changes, we examined how body mass affects five key life-history traits at the individual level: adult survival, breeding probability, breeding success, chick mass and juvenile survival. We found that male mass impacted all traits examined except breeding probability, whereas female mass affected none. Adult male survival increased with increasing mass. Increasing adult male mass increased breeding success and mass of sons but not of daughters. Juvenile male survival increased with their chick mass. These results suggest that a higher investment in sons by fathers can increase their inclusive fitness, which is not the case for daughters. Our study highlights sex-specific differences in the effect of body mass on the life history of a monogamous species with bi-parental care.

## 1. Introduction

Body mass is an important component of an organism's body condition because it reflects the intrinsic amount of energy reserve available to survive and breed [1]. Yet body masses of numerous species have shifted with climate change [2–4]. Therefore, investigating how body mass shapes life-history traits is critical to ultimately understand the impact of environmental changes on individual fitness and population dynamics [5–7].

Body mass and other indices of body condition positively impact adult survival in several species of mammals [5,8–10], and in some long-lived birds such as geese and seabirds [11–13], although sometimes the relationship is only apparent in extreme climatic conditions [14]. This is because long-lived species are expected to allocate their resources primarily to survival rather than reproduction [15]. For example, in poor environmental conditions, yellow-nosed albatrosses (*Thalassarche chlororhynchos*) were only slightly lighter, experienced no change in survival but substantially decreased their provisioning rate to their offspring [16]. Similarly, southern fulmars (*Fulmarus glacialoides*) exposed to poor environmental conditions had lower breeding success, while survival of adults did not vary significantly [17]. This allocation strategy may cause body mass to impact reproductive performances but not survival. Thus, a relationship between body mass and survival is not trivial in long-lived species.

In species with sexual size dimorphism, life-history traits can be expected to respond differently to male and female body mass. The larger sex may suffer costs associated with growth and maintenance of a large size [18–20]. Thus, its life-history traits can be expected to be more sensitive to body mass than the smaller sex, which has lower energetic requirements. Furthermore, sex-specific relationships can arise from segregation in diet choice and space utilization determined by size [21,22] (but see [23]). For example, sexual dimorphism could reduce food competition between sexes in predatory birds as males and females hunt prey related to their respective size [24].

Arguably, the life-history traits most expected to respond differently to male and female body mass are those linked to reproductive performance, even in species providing bi-parental care. This is perhaps straightforward in species where each parent performs a role suited to its size. In predatory birds, where the female is typically the larger sex, large size confers higher reproductive performance to females, whereas it is disadvantageous for males [24–26].

Less obvious are cases of species performing similar parental role, parental contribution and/or parental investment. Parental role here refers to the tasks performed by each parent during reproduction. It is more or less equally partitioned depending on whether the tasks are performed by one or both parents. Parental investment refers to the cost to the parent (i.e. the amount of energy spent by a parent during reproduction). Parental contribution refers to the benefit to the offspring from each parent (i.e. the amount of energy it received from each parent). In the wandering albatross (*Diomedea exulans*), partners incubate and feed their single chick in turns. Thus, parental roles are roughly equally partitioned. However, when rearing chicks, fathers contribute in absolute terms more energy to the chick [27], resulting in unequal parental contribution. Therefore, reproductive performance may respond predominantly to the body condition of the parent contributing the most to reproduction, even in species with bi-parental care.

Here, we examined the effects of adult and chick mass on a suite of life-history traits in the wandering albatross, a long-lived species providing bi-parental care with sexual size dimorphism. Given that this species has increased in mass over the past decades in parallel with an increase in wind speed [28], it is valuable to investigate the effect of mass on individual life-history traits to understand the consequences of environmental changes. Using data from a long-term individual-based study, we studied five life-history traits: adult survival, breeding probability, breeding success, chick mass and juvenile survival.

We predicted that adult mass should increase breeding probability, breeding success and chick mass, but not adult survival, and that chick mass should increase juvenile survival, with possible sex-specific differences. Despite equal role partitioning, given the larger contribution of males during reproduction [29], a more pronounced effect of adult male body mass on reproductive performance was expected.

## 2. Material and methods

### (a) Data collection

The wandering albatross population of Possession Island (Crozet Archipelago), ranging from 250 to 500 breeding pairs, was monitored annually since 1966. The colony is visited multiple times during the breeding season to assess status of individuals [30]. They were classified as chicks (from hatching to departure from the colony), immature (have never bred), non-breeder (have breeding experience but are not breeding during the year of observation), pre-breeder (have breeding experience and are at the colony at the beginning of the breeding season), incubating or chick rearing. Individuals were weighed in 18 different years between 1988 and 2013 in varying status, and in some cases tarsus length was also measured. For a complete description of the field methods, see [31].

#### (i) Standardization of mass

We chose to focus on body mass rather than another index of body condition because (i) measures of structural size, such as tarsus length, were available for only a subset of the individuals with known mass, and (ii) for birds, body mass is a reliable index of body condition [1]. This was also the case in the wandering albatross; a common body condition index, the residuals of a generalized additive model of mass as a function of tarsus length, age and sex of incubating individuals, was strongly correlated to body mass (Pearson correlation coefficient = 0.94 for males and 0.88 for females). The same test for chicks showed the same pattern (Pearson correlation coefficient = 0.94 for males and 0.95 for females). The very strong correlation between the two metrics suggests that they contain the same information.

Mass is a plastic trait that can vary across time, status and age [31]. Thus, mass measurements collected at different points in time are not directly comparable. Prior to performing life-history trait analyses, we built two mass standardization functions, one for adults and another one for juveniles. These enabled us to standardize mass at a reference status relevant for each life-history trait. The standardization functions are presented in detail in the electronic supplementary material, §1.

### (b) Life-history traits and mass

#### (i) Adult and juvenile survival

Mass measurements were available for 662 adults with breeding experience aged between 6 and 30 years (i.e. before the onset of senescence in survival [31,32]); excluding the minor set of senescent individuals and focusing on the majority of the breeding population led to more robust models. Mass measurements were standardized within the year they were taken to the non-breeding mass using the adult mass standardization function as mortality is assumed to occur during the non-breeding period.

The survival analyses for both adults (model set S) and juveniles (model set SJ) were performed in a capture–mark–recapture framework in the program E-Surge to account for imperfect detection [33]. Mass was considered to affect only adult survival from the year of measurement to the next. As the wandering albatross is a facultative biennial breeder, successive capture events are not independent, thus we implemented an immediate trap effect on capture model [34] (more details in the electronic supplementary material, §2).

For the juvenile survival analysis, we distinguished juveniles (individuals aged 1–2 years old never visiting the colony) from the other immatures (individuals at least 3 years old that have not yet bred but can potentially visit the colony). Individuals were grouped into three age classes (1–2, 3–8 and greater than 8 years), following [35]. We examined the effect of chick mass on survival for age 1–2 (referred to as juvenile survival), and allowed age-class-specific and sex-specific observation probabilities.

For each survival analysis, we compared 10 models: (1, 2) two without mass and (3–10) eight with mass. The effect of mass was either (3, 4) distinct for each sex, (5, 6) the same for both sexes, or applied only to (7, 8) males or only to (9, 10) females. Models were constructed with (1, 3, 5, 7, 9) or without (2, 4, 6, 8, 10) a different intercept for each sex on the transition of interest. All continuous variables were standardized. The other transitions were sex- and mass-independent. Models were ranked based on their AICc. Only the model with the lowest AICc and the models within 2ΔAICc that contained no more variables than the previous one were retained following the principle of parsimony [36]. The same model selection procedure has been applied to the other analyses.

#### (ii) Breeding probability

Here, breeding probability is conditioned upon the presence on the island as only present individuals can be weighed. This distinction is important because most individuals skipping breeding do not visit the island [37]. Mass measurements were available for 356 breeders and 55 non-breeders with breeding experience, and were standardized within the year they were taken to the non-breeding mass because non-breeders do not have a breeding mass the years they do not reproduce.

Three breeding probability analyses were performed. The first examined the effect of mass (model set PB). We fitted six generalized linear mixed effect (GLME) models with a binomial distribution and a logit link function including year as a random effect with the package lme4 (v. 1.1–7) [38]. Only one randomly selected entry per individual was kept as the full model did not converge with individual ID as a random effect due to the low number of repeated individuals. To account for the effect of age, we built on the model retained by Pardo *et al*. [32]; consequently, our simplest model included a linear age effect in interaction with sex. We compared this model with a series of age-and-mass models. These models included mass with or without an interaction between mass and sex, or with or without an interaction effect between age and mass, or with or without a three-way interaction between mass, age and sex. Comparison of all possible combination of variables was performed with the ‘dredge’ command from the MuMin package (v. 1.13.4) [39] on models fitted with maximum likelihood. The estimates reported are from the selected models fitted with restricted maximum likelihood.

Because sons are more demanding to rear [27], we additionally tested for a potential impact of fledging a son versus a daughter on male breeding probability the year immediately after the reproductive event (model set PB*_y_*_+1_) and the following year (model set PB*_y_*_+2_). We fitted GLME models with a binomial distribution and a logit function with ID as a random effect. In addition to sex of the previous chick, we considered age of father as a potential explanatory variable.

#### (iii) Breeding success

In 116 cases, mass measurements of both partners of known age and breeding success were available (model set BS). Often, the mass of only one partner was known. Thus, we also examined exclusively the effect of female mass (274 cases; model set BSF) and exclusively of male mass (283 cases; model set BSM). Mass was standardized to the first day of incubation.

We fitted GLME models with a binomial distribution and a logit link function with year and ID as random effects. The minimum model included linear and quadratic effects of age in interaction with sex [32]. We compared the minimum model with age-and-mass models. These models included a combination of an interaction between mass and sex, an interaction between mass and age, or a three-way interaction among mass, age and sex.

#### (iv) Chick mass

Mass measurements of at least one of the parents and of the chick were taken the year of hatching for 89 sexed chicks. Because mass of both parents was not always available, the same statistical approach was performed on three datasets: with both parents (model set MaP, 40 cases), with only the mother (model set MaF, 62 cases) and with only the father (model set MaM, 67 cases). Linear mixed effect models with a Gaussian distribution and year as a random effect were fitted with the lme4 package (v. 1.1–7) [38]. The models compared included a combination of sex of the chick, mass of one or both parents and the interaction between the variables.

## 3. Results

**Figure 1.**
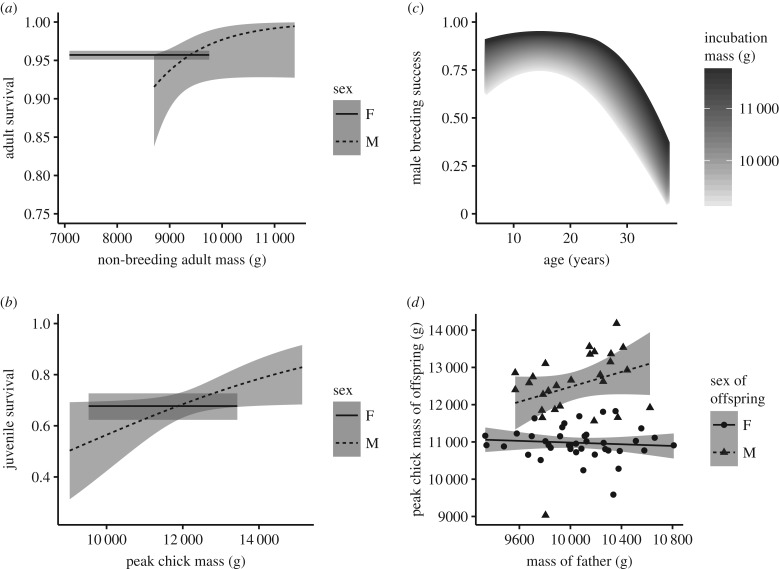
Effect of mass on life-history traits. Predicted annual male and female survival for (*a*) adults based on model S1 and for (*b*) juveniles based on model SJ1. The annual breeding success of males as predicted by the BSM1 is plotted in (*c*) as function of age and mass. The shade gradient represents the effect of mass; confidence intervals in this case have been omitted to improve readability. Peak chick mass of offspring in relation to the mass of father is depicted in (*d*). The dots are the observed values and lines the predicted values. The grey ribbons are the 95% CIs (except for *c*) and have been calculated with the delta method for (*a,b*).

Male adult survival was positively influenced by mass, whereas this was not the case for females (model S1 in tables 1 and 2 and figure 1*a*). The set of models investigating a potential effect of mass on breeding probability found that breeding probability was mass-independent in both sexes as mass was never retained during model selection (model PB1 in table 1). Breeding probability was also independent of the sex of the previous chick, both 1 and 2 years after the last breeding event (models PB1_(*y*+1)_ and PB1_(*y*+2)_ in table 1). A positive effect of mass on breeding success was detected in the model fitted to the dataset including only male mass. Conversely, no effect of mass was detected in the models fitted on the datasets including mass of both partners or only female mass (models BS1, BSF1 and BSM1 in tables 1 and 2 and figure 1*c*).

**Table 1.** Retained models for adult survival (model set S), breeding probability (model sets PB, PB_(*y*+1)_ and PB_(*y*+2)_), breeding success (model sets BS, BSF and BSM), chick mass (model sets Ma, MaF and MaM) and juvenile survival (SJ). Model set PB tested for the effect of mass on breeding probability, model set PB_(*y*+1)_ for the effect of previous-year offspring sex, model set PB_(*y*+2)_ for the effect of sex of offspring two years previous. Model sets BS, BSF and BSM are performed on different datasets: including both partners, only females and only males, respectively. The same applied to model sets testing for an effect of adult mass on chick mass: including both partners (Ma), only females (MaF) and only males (MaM). If not specified, mass refers to adult mass. AICc values are estimated from models fitted by ML. Within each model set, only the model with the lowest AICc (ΔAICc = 0) and the models with fewer parameters within 2ΔAICc from the model with ΔAICc = 0 are reported. The ΔAICc is the difference in AICc from the model with the lowest AICc within the model set. For the survival analyses, d.f. refers to the number of estimable parameters; it adds up to more than the number of explanatory variables listed because it also includes the parameters for the probability of recapture (not listed because they were identical in all models). For the other models, d.f. refers to the degree of freedom for linear mixed effect models based on the inner–outer rules [40].

model set	model	resp. var	expl. var	d.f.	AICc	ΔAICc
adult survival						
S	S1	*sur.* ∼	*sex + mass (M)*	8	8940.68	0
	S2	*sur.* ∼	*mass (M)*	7	8941.65	0.97
breeding probability						
PB	PB1	*prob. bre.* ∼	*sex + age + sex : age*	5	260.87	0
PB_(*y*+1)_	PB1_(*y*+1)_	*prob. bre.*_(*y*+1)_ ∼	*age*	3	502.8	0
PB_(*y*+2)_	PB1_(*y*+2)_	*prob. bre.*_(*y*+2)_ ∼	*age*	3	2244.5	0
breeding success						
BS	BS1	*bre. suc.* ∼	*age (F) + age^2^ (F) + age (M) + age^2^ (M)*	7	122.83	0
BSF	BSF1	*bre. suc.* ∼	*age + age^2^*	5	321.59	0
BSM	BSM1	*bre. suc.* ∼	*age + age^2^ + mass*	6	294.38	0
	BSM2	*bre. suc.* ∼	*age + age^2^*	5	294.76	0.38
chick mass						
Ma	Ma1	*chick mass ∼*	*sex_chick_*	4	652.56	0
MaF	MaF1	*chick mass ∼*	*sex_chick_ + age*	5	997.51	0
	MaF2	*chick mass ∼*	*sex_chick_*	4	997.83	0.33
MaM	MaM1	*chick mass ∼*	*sex_chick_ + mass + mass : sex_chick_*	6	1074.59	0
	MaM2	*chick mass ∼*	*sex_chick_*	4	1075.01	0.42
juvenile survival						
SJ	SJ1	*chick* *sur.* ∼	*age (1–2) + age (3–8) + age (>8) + mass_chick_ (M)*	8	2981.00	0

**Table 2.** Estimates on the logit scale of the coefficients of the fixed effects included in the models in table 1 fitted by REML. *1st trans.* refers to the yearly survival from the first year after mass measurement and *subs. trans.* to all the subsequent yearly survival.

model	resp. var	expl. var	estimate	s.e.
S1	*sur.*∼	*intercept for 1st trans. (F)*	3.106	0.072
		*intercept for 1st* *trans*. *(M)*	2.759	0.321
		*subs*. *trans*.	1.791	0.434
		*mass (M)*	1.078	0.583
S2	*sur*.∼	*intercept for 1st trans. (F)*	3.105	0.072
		*intercept for 1st trans. (M)*	3.681	0.666
		*subs. trans.*	1.913	0.421
PB1	*prob*. *bre*.∼	*intercept (F)*	2.700	0.694
		*sex (M)*	−0.147	0.364
		*age*	−0.133	0.298
		*sex (M) : age*	0.151	0.398
	*marg. R^2^: 0.02, cond. R^2^* *:* *0.60, rand eff.: year*			
BS1	*bre*. *suc*.∼	*intercept*	2.338	0.603
		*age (M)*	0.529	0.498
		*age^2^ (M)*	−0.871	0.324
		*age (F)*	−0.420	0.582
		*age^2^ (F)*	0.076	0.250
	*marg. R^2^: 0.25, cond. R^2^: 0.34, rand eff.: year* *+* *ID*			
PB1_(y+1)_	*prob*. *bre*._(y+1)_ ∼	*intercept*	−8.15	0.68
		*age*	0.47	0.19
	*marg. R^2^: 0.00, cond. R^2^: 0.00, rand eff.: ID*			
PB1_(y+2)_	*prob*. *bre*._(y+2)_ ∼	*intercept*	0.86	0.05
		*age*	−0.08	0.05
	*marg. R^2^: 0.00, cond. R^2^: 0.03, rand eff.: ID*			
SF1	*bre*. *suc*. (F)∼	*intercept*	1.253	0.233
		*age*	0.172	0.196
		*age^2^*	−0.129	0.109
	*marg. R^2^: 0.01, cond. R^2^: 0.06, rand eff.: year + ID*			
BSM1	*bre*. *suc*. (M)∼	*intercept*	1.645	0.247
		*age*	−0.182	0.216
		*age^2^*	−0.312	0.129
		*mass*	0.307	0.193
	*marg. R^2^: 0.13, cond. R^2^: 0.16, rand eff.: year+ID*			
BSM2	*bre*. *suc*. (M)∼	*intercept*	1.740	0.266
		*age*	−0.044	0.199
		*age^2^*	−0.378	0.126
	*marg. R^2^: 0.12, cond. R^2^: 0.17, rand eff.: year* *+* *ID*			
MaP1	*mass_chick_* ∼	*intercept (F)*	10 997	157.7
		*sex_chick_ (M)*	1324.2	249.3
	*marg. R^2^: 0.42, cond. R^2^: 0.42, rand eff.: year*			
MaF1	*mass_chick_* ∼	*intercept (F)*	11 368.11	230.25
		*sex_chick_ (M)*	1246.83	178.66
		*age*	142.64	87.25
	*marg. R^2^: 0.40, cond. R^2^: 0.55, rand eff.: year*			
MaF2	*mass_chick_* ∼	*intercept (F)*	11 352.34	228.6
		*sex_chick_ (M)*	1236.66	181.12
	*marg. R^2^: 0.38, cond. R^2^: 0.52, rand eff.: year*			
MaM1	*mass_chick_* ∼	*intercept (F)*	10 958.32	172.28
		*sex_chick_ (M)*	1512.05	167.86
		*mass*	−42.47	100.7
		*mass : sex_chick_*	367.5	177.19
	*marg. R^2^: 0.54, cond. R^2^: 0.59, rand eff.: year*			
MaM2	*mass_chick_* ∼	*intercept (F)*	10 970.98	174.71
		*sex_chick_ (M)*	1505.86	171.79
	*marg. R^2^: 0.51, cond. R^2^: 0.57, rand eff.: year*			
SJ1	*juv* *sur*. ∼	*intercept for age (1–2)*	0.741	0.122
		*intercept for age (3–8)*	7.791	40.82
		*intercept for age (>8)*	2.737	0.417
		*chick mass (M)*	0.259	0.130

We did not detect any effect of parent mass on chick mass in models fitted to datasets including both parents or only the mother (models MaP,1 MaF1 and MaF2 in tables 1 and 2), whereas father's mass was retained in the top model when using the dataset including only father's mass (model MaM1 in table 2). Father's mass was found to positively impact chick mass in sons, but not in daughters (model MaM1 in table 2 and figure 1*d*). Chick mass had a positive effect on the annual male juvenile survival but not for females (model SJ1 in table 2 and figure 1*b*).

## 4. Discussion

This study provides a comprehensive overview of the effect of body mass on life-history traits of a sexually dimorphic long-lived species providing bi-parental care. We found a clear difference between sexes: male mass enhanced performance in four life-history traits, namely adult survival, breeding success, chick mass and juvenile survival, whereas female mass impacted none. Sex-specific relationships between mass and life-history traits were expected, given the different ecology of the two sexes. Indeed, males and females forage in different oceanic sectors [41], follow a different relationship between wind, mass and foraging statistics such as flight speed and maximum distance from the colony [42], and contribute differently to reproduction [27,29].

### (a) Effect of mass on survival

We found that body mass of adult males enhances survival in one of the longest-lived birds, irrespective of environmental conditions. This is not trivial as long-lived species are expected to allocate resources to survival before reproduction [15], which can potentially buffer an influence of mass on survival even in extremely poor environmental conditions. For instance, there is no such relationship in the blue petrel (*Halobaena caerulea*) [43] and the lack of variation in survival in the southern fulmar, even in poor environmental conditions, suggests likewise [17]. Yet the case of the wandering albatross is not unique. Shorter-lived seabirds—the little auk (*Alle alle*), in extreme environmental conditions [14], and the black-legged kittiwake (*Rissa tridactyla*) [11,44]—also had enhanced survival when in good body condition.

Unlike male survival, female adult survival of wandering albatrosses was not impacted by body mass. Such a difference may reflect the higher energetic requirements of the larger sex to maintain its large size [19,20] or may be related to sex-specific energetically demanding activities. For example, in the greater white-fronted goose (*Anser albifrons*), only female survival varies with body mass, which is explained by the higher energetic requirement of this sex, the only one that incubates [13]. Similarly, male wandering albatrosses experience male-specific energetically demanding periods in their life cycle, in particular during the pre-breeding period when they are guarding the nest for one month without feeding, and at the end of the breeding season when they provision for the chick for longer than females [45].

The contrasted relationships between mass and survival among sexes may have important consequences for population dynamics. Indeed, they can affect population growth rate directly and indirectly through the population structure, operational sex ratio (OSR) and mating process [46]. For example, male emperor penguin survival is determined by environmental conditions while female survival is not [47], which impacts OSR. The sensitivity of the population growth rate to female survival is negative [46] as the indirect effects through OSR can sometimes overwhelm the direct effects. For the wandering albatross, the consequences on population growth rate of the contrasted adult survivals remain an open question.

### (b) Mass-independent breeding probability

As in the blue petrel [43], breeding probability of adult wandering albatrosses was not influenced by body mass. This is intriguing because body mass determines the first breeding attempt: among individuals of the same age, immature birds tend to be lighter than breeders [31]. It is possible that the absence of an effect of body mass on breeding probability is an artefact. Breeding probability examined here is conditional upon return to the colony. The non-breeders sampled may have returned because they were in good enough condition to breed, but some failed to breed for reasons independent of their own condition, for instance, because their partners were absent. Birds in poor condition may simply have not returned to the colony and remain at sea. We cannot test for this assumption, as individuals that are absent from the colony cannot be weighed.

### (c) Effect of mass on reproductive performance

Reproductive performance varied with the body mass of the father, the parent contributing the most to reproduction [29], but not with that of the mother. The dependence on exclusively one parent can be linked to how parents resolve conflict over offspring care. In a bi-parental system with perfect information, parents should incompletely compensate for a reduction in their partner contribution when increasing contribution yields decelerating benefits to the offspring at a non-decelerating cost to the parent [48]. That is, when the costs to the parent are high and the benefit to the offspring low, the parent has little incentive to increase investment. When, from the same level of cost, the contribution to reproductive effort (i.e. the benefit to the offspring) differs between the parents, the parent with the lowest contribution for the same cost can compensate less than the other parent. This is possibly the case in the wandering albatross.

The absence of a relationship between wandering albatross female mass and either breeding success or chick mass probably does not reflect a lack of investment of females. Rather, it indicates that no matter the condition of the female, her absolute contribution to reproduction cannot compensate for a reduced contribution of the male. Females probably bear costs proportional to those of males, as suggested by the parallel mass variation of males and females during chick rearing [29]. Furthermore, in proportion to their mass, females provide larger meals—even though they visit the nest less often [27]. Yet, for the same mass, the wing loading of females, which have smaller wing surface, changes more than that of a male, suggesting that the energetic cost of females per meal is larger as higher wing loading increases the cost of taking off and landing [49]. Indeed, in the wandering albatross population of South Georgia, there is evidence that female cost of chick rearing is higher than that of males [50]. Similarly, in the northern giant petrel, a seabird with a sexual size dimorphism even more pronounced than the wandering albatross, female foraging costs are much higher than those of males [22]. It thus is probable that females, the smaller sex, do not invest less, but that their contribution has less impact.

Unequal contribution to reproduction is not uncommon in bi-parental systems. Other seabirds show higher food provisioning from one parent, usually the largest sex [51,52], but also in the absence of sexual size dimorphism [53,54]. In species like the wandering albatross where there is an asymmetry in contribution between parents, the reproductive performance can be expected to depend more on the condition of the parent contributing the most.

### (d) Fathers invest more in sons

Numerous previous studies that experimentally manipulated body conditions of the parents (or one parent) and/or chick need reported parents’ (or one parent's) ability to adjust food provisioning to both chick and parents conditions (e.g. [46,48]). However, here we found evidence that this is the case in an unmanipulated system. By examining jointly the effect of adult body mass on chick body mass and the effect of chick body mass on juvenile survival, we could detect indirect benefits to the adult of investing more in a chick to improve its post-fledging survival.

The strategy of wandering albatross fathers is consistent with the current theory stating that long-lived seabirds should adjust their reproductive performance to both their own body condition and the need of their offspring [55]. We found evidence of higher investment of fathers in sons, but not in daughters with increasing father mass. This trend corroborates well with the higher cost of rearing sons observed in many species [56–58]. In the wandering albatross, both parents provide more food to sons than to daughters, sons have faster growth rates and reach higher fledgling mass [27], and parents adjust meal size to male chick needs [29]. The higher energetic need of males is often explained by the higher vulnerability of male juveniles to food shortage compared with females [18], a hypothesis likely to be applicable to the wandering albatross as male juveniles have a lower survival ([35], this study).

Our results suggest that, in wandering albatrosses, investing further in sons to respond to their higher energy requirement is an efficient strategy for fathers to increase their inclusive fitness because it increased sons' juvenile survival, and it came at no cost to future breeding performance. Indeed, heavy male chicks had a higher juvenile survival than lighter ones, whereas mass had no effect on female juvenile survival. Furthermore, the breeding probability of fathers that produced sons 1 or 2 years later was not lower than that of fathers that produced daughters. This suggests that producing a son has no carry-over effect on male breeding performance in the short term. Not only do fathers adjust investment in their sons to their sons' conditions [29], but also to their own. Fathers seem to share with their sons only the resources that they can spare within a given year.

## 5. Conclusion

Our results showed that among long-lived species, a relationship between body mass and survival can be present independently of environmental conditions. The survival of only one of the two sexes can be affected by body mass but not the other, probably due to sex-specific energy requirements [19]. Furthermore, we showed that life-history traits related to reproduction of species with sexual size dimorphism with bi-parental care can vary exclusively with body condition of the sex contributing the most to reproduction. Theory on resolution of parental conflict over care provides a framework to explain the emergence of such a pattern [55]: when, for the same cost to the parents, the benefits derived by the offspring produced from the care of each parent are unequal, then the parents contributing the less may not be able to compensate.

When some life-history traits are mass-dependent, variation in mass distribution is expected to have consequences at population level. Given that body mass has been reported to have changed over the past years with wind speed in both sexes [28], understanding how a climate-driven change in this trait will affect population dynamics will prove to be crucial for the conservation of this species.

## Supplementary Material

SI1 Mass standardization functions

## Supplementary Material

SI2 Adult survival

## Data accessibility

Data will be made available on Dryad.
