# Supplementary material for: Fathers matter: male body mass affects life-history traits in a size-dimorphic seabird
Source: Proc Biol Sci. 2017 May 3;284(1854):20170397. doi: 10.1098/rspb.2017.0397 (PMC5443952; doi:10.1098/rspb.2017.0397)
Supplement: SI1 Mass standardization functions [file rspb20170397supp1.pdf]

Supplementary Information

SI1 Mass standardization functions

Adult mass

The adult mass standardization function describes adult mass as a function of sex, age, year, status, and number of days into breeding stage for pre-breeding, incubation and chick rearing. Unless specified otherwise, analyses were performed in R (version 3.0.2, [1]). A generalized additive mixed model (GAMM) was fitted with the package mgcv (version 1.8-6, [2]) to account for nonlinearity with full tensor product smooths for fixed continuous effects and with individual ID and year as a random effects.

Chick mass

Between 1999 and 2008, 412 sexed chicks were weighed around the period of peak chick mass [3]. We built a chick mass standardisation function to estimate mass at a given day of the year by fitting a GAMM with chick mass as dependent on sex, day of year and year of birth as fixed effects with full tensor product smooths for the day of the year, and chick ID as a random effect. Chick mass was standardised to the 30th September of their birth year, the date at which most mass measurements were taken.

SI Table 1: The estimates of the categorical factors of the GAM used to **standardize adult mass**.

	Estimate	SE	t	p-value
Intercept (chick rearing, F)	7752.92	317.61	24.41	
Year 1989	-1129.17	276.41	-4.09	<0.001
Year 1990	-878.33	270.73	-3.24	0.001
Year 1991	-996.99	644.63	-1.55	0.122
Year 1992	-800.43	281.31	-2.85	0.005
Year 1998	141.46	292.78	0.48	0.629
Year 1999	-976.36	289.99	-3.37	0.001
Year 2000	-340.58	565.84	-0.60	0.547
Year 2001	-387.63	279.28	-1.39	0.166
Year 2002	-649.13	291.68	-2.23	0.026
Year 2003	-674.87	275.34	-2.45	0.014
Year 2004	-570.66	307.38	-1.86	0.064
Year 2005	163.49	379.51	0.43	0.667
Year 2008	-502.92	332.03	-1.52	0.130
Year 2009	9.36	325.54	0.03	0.977
Year 2011	-749.56	300.28	-2.50	0.013
Year 2012	-420.37	314.11	-1.34	0.181
Year 2013	-622.41	300.01	-2.08	0.038
Sex (M)	1762.54	61.91	28.47	<0.001
Incubating	1098.48	192.02	5.72	<0.001
Not breeding	962.62	233.34	4.13	<0.001
Pre-breeding	1459.91	326.97	4.47	<0.001
Immature	-1794.19	1872.04	-0.96	0.338

SI Table 2: The estimates of smooth functions of the continuous predictors and the random effect of the GAM used to standardize adult mass. The abbreviations adu stands for adult, imm for immatures, inc. for incubation, nbr for number and rear. for rearing.

	Edf	Ref.df	F	p-value
Age (Adu)	2.25	2.64	7.65	<0.001
Age (Imm)	1.90	1.97	6.42	0.002
nbr of days into inc.	3.50	3.85	11.46	<0.001
nbr of days into chick rear.	2.28	2.58	12.43	<0.001
ID (random effect)	260.78	648.00	0.70	<0.001

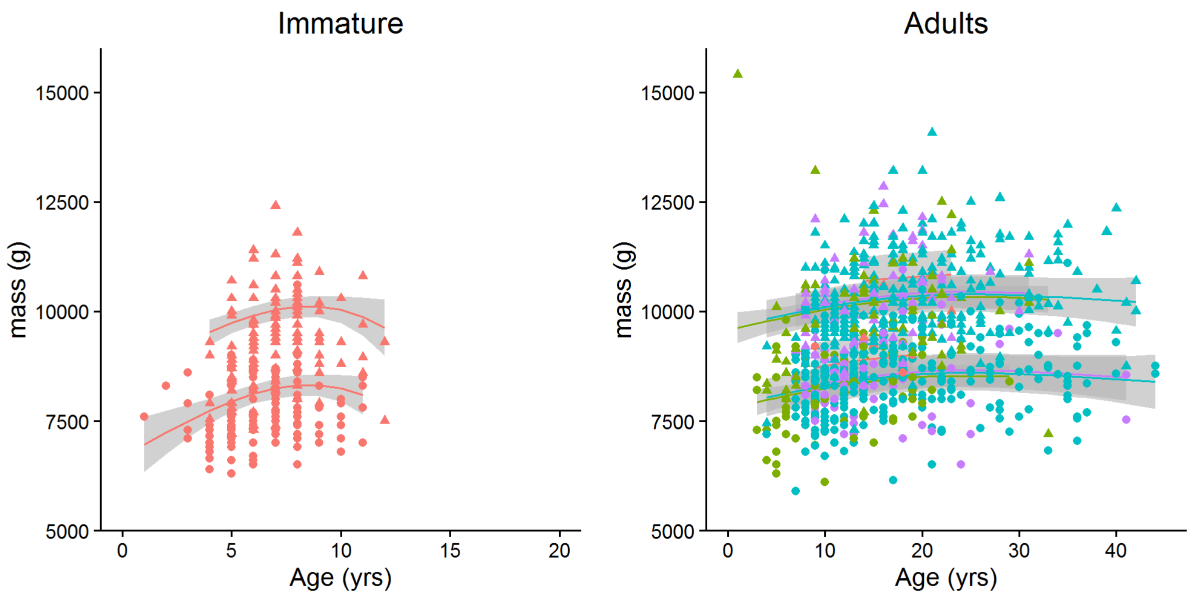

SI Figure 1: The relationship between age and mass as predicted by mass standardization function. On the right graph is the relationship for adults and on the left for immature birds. Points are original data: triangles for males and circles for females. In the right graph, the different colours reflect the state of the individual: pre-breeder are in orange, non-breeder in green, incubating individuals are in blue and chick rearing breeders are in green. Predicted values (lines) have been estimated for year 2001 at the first day of incubation, and chick rearing respectively.

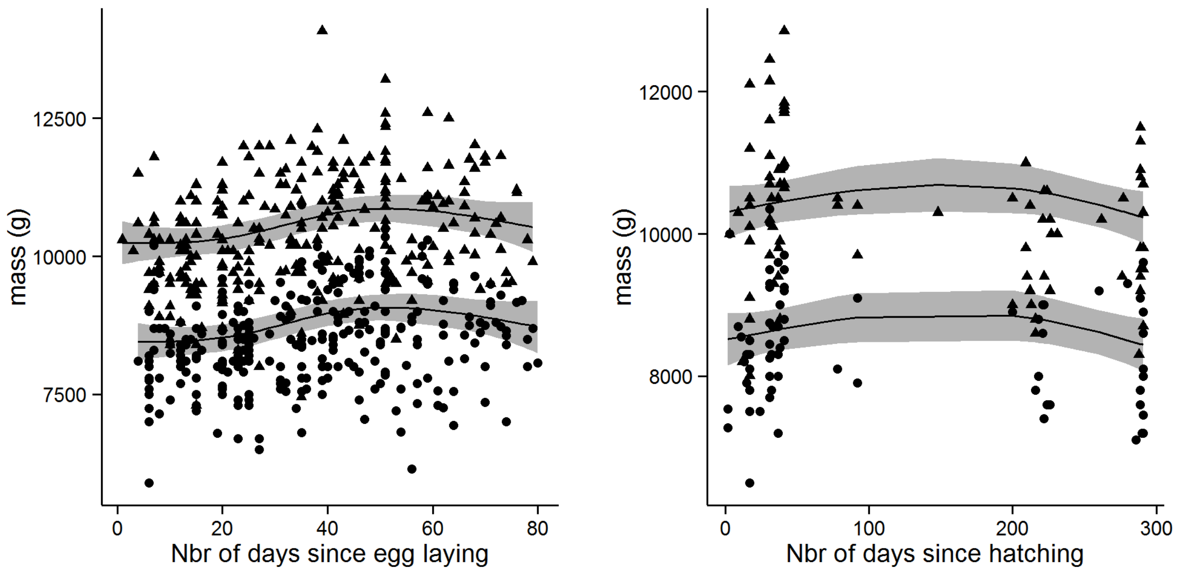

SI Figure 1: The relationship between mass and the number of days into incubation (left) and number of days into chick rearing (right) as predicted by the mass standardization functions. Points are observed data: triangles for males and circles for females. Predicted values (lines) have been estimated for year 2001 and for mean age (14 years old). The grey shaded area is the 95% confidence interval.

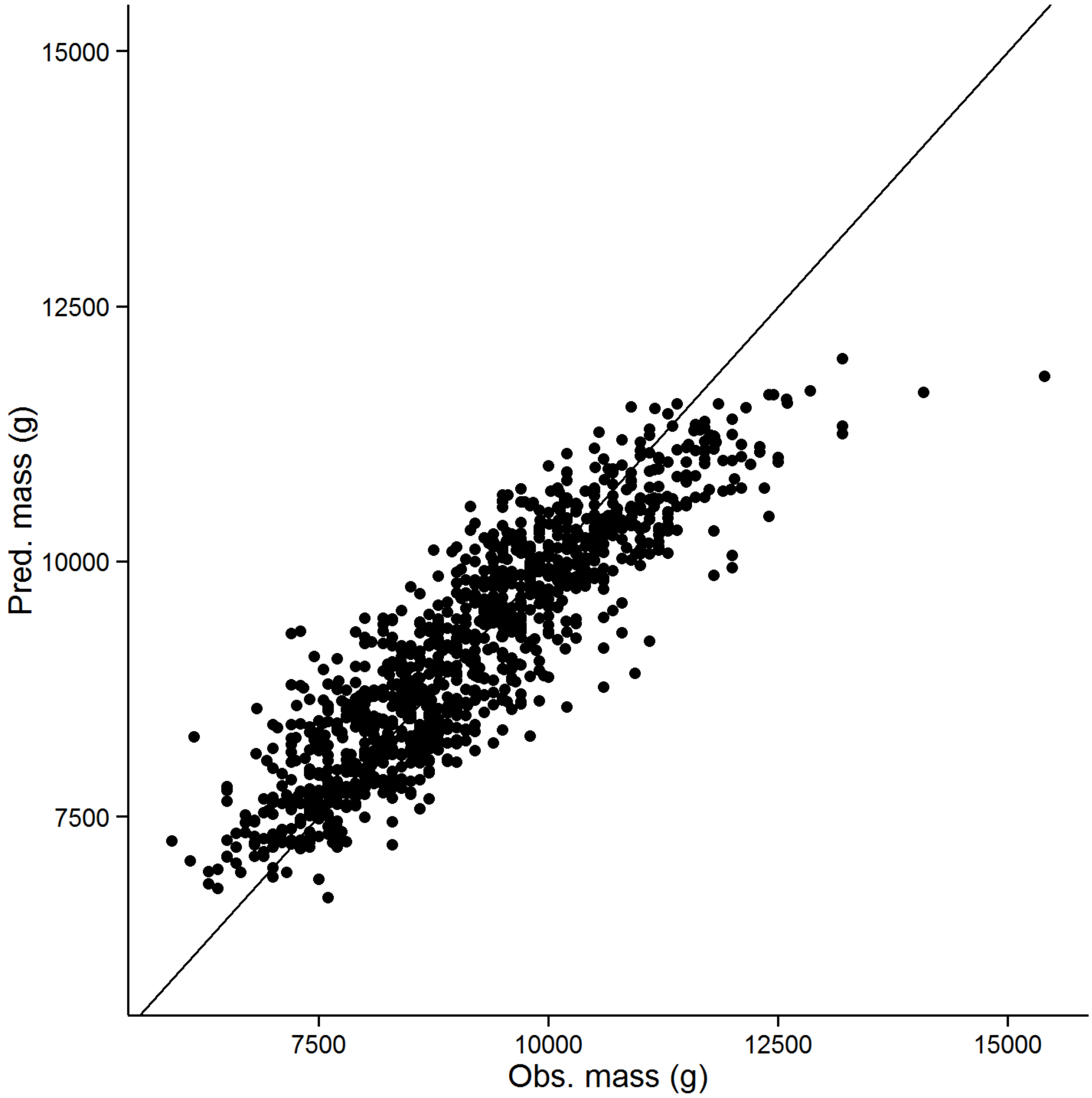

SI Figure 2: Relationship between predicted mass and observed mass as based on the model to standardize mass. The line is the 1:1 line.

SI Table 3: The estimates of the categorical factors of the GAM used to **standardize chick mass.**

	Estimate	SE	t	p
Intercept	10583.83	170.79	61.97	<0.001
Sex(M)	1530.07	129.26	11.84	<0.001
Year 2000	-99.97	381.72	-0.26	0.794
Year 2001	16.38	383.33	0.04	0.966
Year 2002	795.93	321.87	2.47	0.014
Year 2003	-86.11	199.60	-0.43	0.666
Year 2004	80.87	260.61	0.31	0.757
Year 2005	490.07	258.34	1.90	0.059
Year 2006	2108.48	631.48	3.34	0.001
Year 2007	212.32	258.95	0.82	0.413
Year 2008	-36.42	282.96	-0.13	0.898

SI Table 4: The estimates of smooth functions of the continuous predictors and the random effect of the GAM used to standardize chick mass.

	edf	Ref.df	F	p-value
Days since beginning of the year	3.521	3.77	3.52	<0.001
ID (random effect)	159.25	401	159.26	<0.001

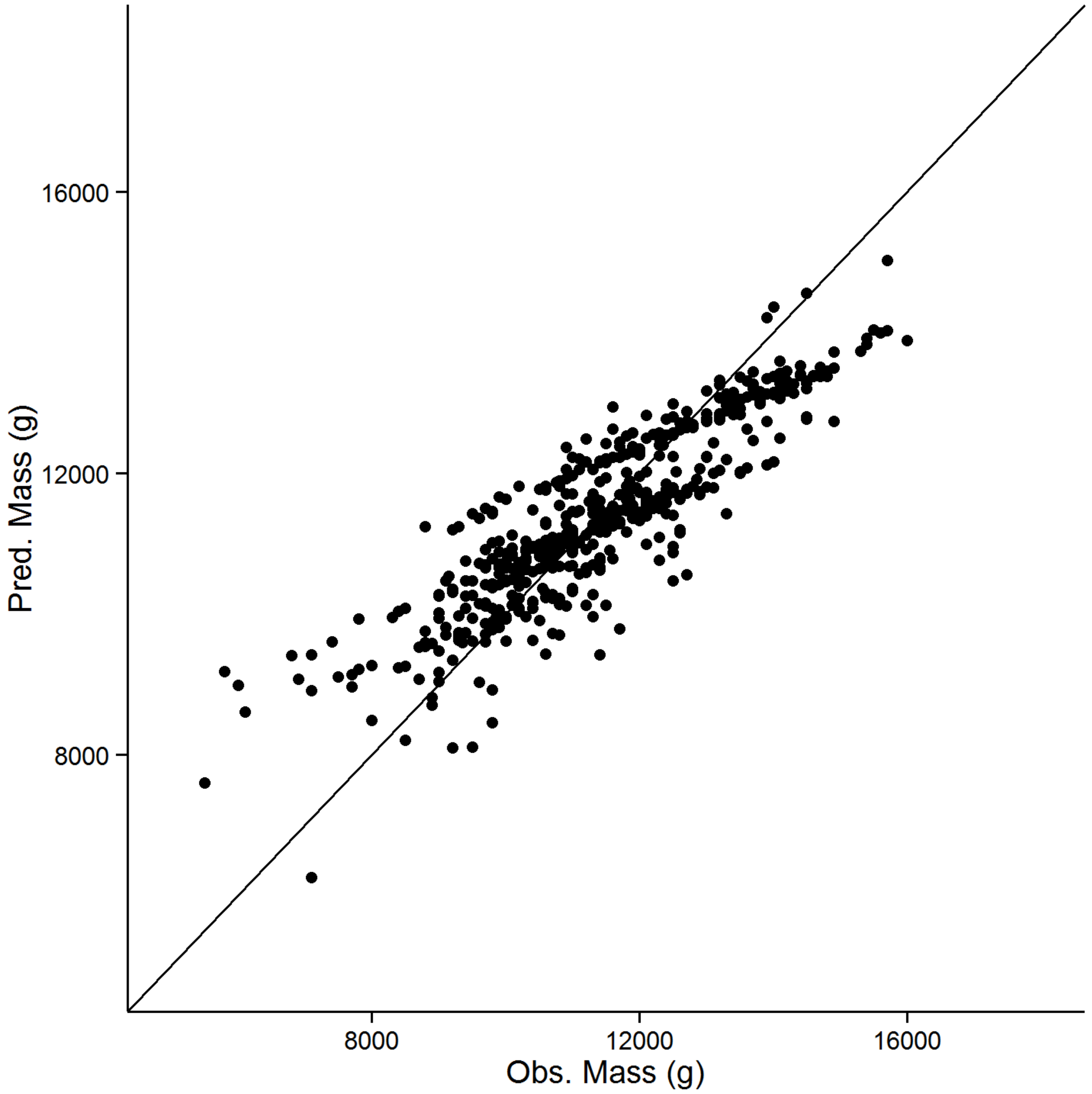

SI Figure 3: Relationship between predicted and observed chick mass as based on the model to standardized chick mass. The line is the 1:1 line.

Literature

1. R Core Team 2016 R: A language and environment for statistical computing. *R Dev. Core Team*.

2. Wood, S. N. 2011 Fast stable restricted maximum likelihood and marginal likelihood estimation of semiparametric generalized linear models. *J. R. Stat. Soc. Ser. B Stat.*

Methodol. **73**, 3–36.

3. Weimerskirch, H. & Lys, P. 2000 Seasonal changes in the provisioning behaviour and mass of male and female wandering albatrosses in relation to the growth of their chick. *Polar Biol.* **23**, 733–744.
