# Supplementary material for: Fathers matter: male body mass affects life-history traits in a size-dimorphic seabird
Source: Proc Biol Sci. 2017 May 3;284(1854):20170397. doi: 10.1098/rspb.2017.0397 (PMC5443952; doi:10.1098/rspb.2017.0397)
Supplement: SI2 Adult survival [file rspb20170397supp2.pdf]

Supplementary Information

SI2 Adult survival

To estimate the effect of mass on adult survival, we modified adult encounter histories such that individuals entered the population the year they were weighed. For example, the encounter history of an individual born in 1990 but weighed in 1999 started only in 1999, even if the individual was re-sighted earlier. This approach allowed individual-based analysis when most individuals are only weighed once throughout their life time.

As the wandering albatross is a non-obligate biennial breeder, successive capture events are not independent. To account for this, we implemented an Immediate Trap Effect on Capture model (ITEC) [1]; individuals are either trap aware if they have been captured at the immediate previous occasion or trap unaware if not. We defined a sex and mass-dependent survival process and sex-specific trap-awareness process depending on the previous state (aware/unaware).

The transition matrix is the multiplication of the survival matrix S_t with the trap awareness process matrix P which takes place between the intermediate time step $t+1^-$ and $t+1^+$. The different stages are A trap aware, U trap unaware (both have the same survival, ϕ) and D for dead. The probability, p differs between the trap aware and trap unaware stages and captures the probability to be encountered and become trap aware. The matrix of event *E*, is trivial, a trap-aware individual has necessarily been seen at the previous occasion. From [1].

$A_{t+1^-} \quad U_{t+1^-} \quad D_{t+1^-}$

$$S_t = \begin{array}{c|ccc|} A_t & \Phi & 0 & 1\text{-}\phi \\ U_t & 0 & \Phi & 1\text{-}\phi \\ D_t & 0 & 0 & 1 \end{array}$$

$$P_{t+1} = \begin{array}{c|ccc|} & A_{t+1^{+}} & U_{t+1^{+}} & D_{t+1^{+}} \\ A_{t+1^{-}} & p' & 1\text{-}p' & 0 \\ U_{t+1^{-}} & p & 1\text{-}p & 0 \\ D_{t+1^{-}} & 0 & 0 & 1 \end{array}$$

$$E_t = \begin{array}{c|cc|} & \text{"0"} & \text{"1"} \\ A_t & 0 & 1 \\ U_t & 1 & 0 \\ D_t & 1 & 0 \end{array}$$

Literature

1. Pradel, R. & Sanz-Aguilar, A. 2012 Modeling trap-awareness and related phenomena in capture-recapture studies. *PLoS One* **7**, 1–4.
